# What to Do with the Jubilee Stamps

**Published:** 1897-06-19

**Authors:** 


### WHAT TO DO WITH THE JUBILEE STAMPS.

Some people are asking what they shall do with the stamps when they have bought them, and it is not an altogether unreasonable question. To stamp collectors no answer is necessary. A stamp like this, the total issue of which is absolutely limited and the plate of which will be destroyed before witnesses when the required number of impressions have been struck off, is a curiosity which every philatelist will desire to have in his collection. We are not all stamp collectors, however, and it is well to bear in mind that there are many ways in which these stamps may be so used as to form both pretty and most acceptable mementoes of many events of which people will be very glad to be reminded in years to come, besides serving the primary function of acting as a receipt for money given in a good cause. First we must mention the Queen's Commemoration Bible and Prayer Book, each copy of which will carry one of the stamps. Then there is the Illustrated Programme of the Jubilee Procession, the proceeds of the sale of which will go to the Prince of Wales's Hospital Fund; in each copy of this there are vacant places intended to be filled up with the stamps. A number of special mounts have been prepared, which, when fitted with the stamps, and especially when framed, form very attractive decorations. Some of these are very neatly embossed in gold with a replica of the signature of the Queen. Frames and mounts for preserving the stamps have been prepared by Messrs. De la Rue. Then there is Lincoln's mount, with the arms of the colonies; also a plain mount with space for a specimen of each stamp. A solid and well-gilt miniature frame, made by Mr. A. Walker, of 118, New Bond Street, can be had for 4s., exclusive of the stamp. It is suggested to us that the stamp would form an admirable decoration to a menu or invitation card if artistically worked in, and in view of the expense that many people go to in these matters, we cannot but think the suggestion a most appropriate one. Many of our colonial friends will carry home with them these little records of the hospitality they have enjoyed, and there is no doubt that an added value would be given to them did they contain one of these stamps, which are commemorative of the charitable aspect of a national celebration.

